# Carotid artery plaque in women with rheumatoid arthritis and low estimated cardiovascular disease risk: a cross-sectional study

**DOI:** 10.1186/s13075-015-0576-7

**Published:** 2015-03-11

**Authors:** Alfonso Corrales, Patrick H Dessein, Linda Tsang, Trinitario Pina, Ricardo Blanco, Carlos Gonzalez-Juanatey, Javier Llorca, Miguel A Gonzalez-Gay

**Affiliations:** Epidemiology, Genetics and Atherosclerosis Research Group on Systemic Inflammatory Diseases, Rheumatology Division, IDIVAL, Avenida Cardenal Herrera Oria s/n, Santander, 39011 Spain; Cardiovascular Pathophysiology and Genomics Research Unit, School of Physiology, Faculty of Health Sciences, University of the Witwatersrand, 7 York Road, Parktown 2193, Johannesburg, South Africa; Cardiology Division, Hospital Lucus Augusti, Calle Dr. Ulises Romero 1, 27003 Lugo, Spain; Department of Epidemiology and Computational Biology, School of Medicine, University of Cantabria, and CIBER Epidemiología y Salud Pública (CIBERESP), IDIVAL, Avenida Cardenal Herrera Oria s/n, 39011 Santander, Spain; School of Physiology, University of the Witwatersrand, 7 York Road, Parktown 2193, Johannesburg, South Africa

## Abstract

**Introduction:**

We previously reported that most patients with rheumatoid arthritis (RA) and moderate cardiovascular disease (CVD) risk according to the Systematic COronary Evaluation score (SCORE) experience carotid artery plaque. In this study, we aimed to identify patient characteristics that can potentially predict carotid plaque presence in women with RA and a concurrent low CVD risk according to the SCORE.

**Methods:**

A cohort of 144 women with an evaluated low risk of CVD (SCORE value of zero) was assembled amongst 550 consecutive patients with RA that underwent CVD risk factor recording and carotid artery ultrasound. Participants had no established CVD, moderate or severe chronic kidney disease, or diabetes. We assessed carotid plaque(s) presence and its associated patient characteristics.

**Results:**

Carotid artery plaque was present in 35 (24.3%) of women with RA. Age, the number of synthetic disease-modifying agents (DMARDs) and total cholesterol concentrations were independently associated with plaque in multivariable stepwise backward regression analysis (odds ratio (95% confidence interval) = 1.15 (1.07 to 1.24), *P* <0.0001, 1.51 (1.05 to 2.17), *P* = 0.03 and 1.66 (1.00 to 2.73) *P* = 0.04), respectively). The area under the curve (AUC) of the receiver operating curve (ROC) for the association with plaque was 0.807 (*P* <0.0001), 0.679 (*P* = 0.001) and 0.599 (*P* = 0.08) for age, total cholesterol concentrations and number of synthetic DMARDs used, respectively. The optimal cutoff value in predicting plaque presence for age was 49.5 years with a sensitivity and specificity of 74% and 75%, respectively, and for total cholesterol concentration, it was 5.4 mmol/l with a sensitivity and specificity of 63% and 70%, respectively. The plaque prevalence was 37.5% in patients (n = 80; 55.6%) with age >49.5 years or/and total cholesterol concentration of >5.4 mmol/l, respectively, compared to only 7.8% in those (n = 64; 44.4%) with age ≤49.5 years or/and total cholesterol concentration of ≤5.4 mmol/l, respectively.

**Conclusions:**

Approximately one-third of women with RA who experience a low SCORE value and are aged >49.5 years or/and have a total cholesterol concentration of >5.4 mmol/l, experience high-risk atherosclerosis, which requires intensive CVD risk management.

**Electronic supplementary material:**

The online version of this article (doi:10.1186/s13075-015-0576-7) contains supplementary material, which is available to authorized users.

## Introduction

Rheumatoid arthritis (RA) is a chronic inflammatory disease that enhances the risk of atherosclerotic cardiovascular event rates to a similar extent as type 2 diabetes [1]. Traditional risk factors do not fully explain the increased cardiovascular disease (CVD) risk in RA [2]. In fact, traditional risk factors and disease characteristics, in particular high-grade inflammation, associate overall additively and as strongly with atherosclerosis and incident CVD event rates in RA [3-5].

The EUropean League Against Rheumatism (EULAR) recently reported recommendations for CVD risk management in inflammatory arthritis including RA [1]. This comprised risk stratification based on the Systematic COronary Evaluation Score (SCORE), a multiple major traditional risk factor equation, together with the use of a multiplier of 1.5 when two of three criteria were met; the latter consisted of a disease duration >10 years, rheumatoid factor or/and anticyclic citrullinated peptide positivity and the presence of severe extra-articular manifestations, thereby providing a modified (m) SCORE in patients with RA. However, the mSCORE can underestimate the actual CVD risk in patients with RA [6,7].

Carotid artery plaque as identified by ultrasound represents very high CVD risk [8,9] and indeed strongly predicts incident cardiovascular event rates in both non-RA and RA subjects [8-10]. Accordingly, the recent European CVD prevention guidelines [8] recommend risk management that includes the use of cardiovascular drugs, as in secondary prevention, with a low-density lipoprotein (LDL) cholesterol target of <1.8 mmol/l, in persons with carotid artery plaque [8]. Carotid ultrasound is particularly indicated in non-RA subjects at moderate risk of CVD [8]. In this regard, we recently documented that amongst RA patients with an mSCORE value of ≥1 and <5 that reflects moderate CVD risk, as many as 63% had carotid artery plaque [11].

Advancing age contributes substantially to calculated mSCORE values in individual persons [1,8] whereas disease characteristics rather than traditional risk factors are reportedly most strongly associated with atherosclerosis, especially in young patients with RA [12]. It is therefore conceivable, and was indeed confirmed, that in patients with RA and a concurrent low predicted cardiovascular event rate as estimated by SCORE, that is, when the respective calculated value is zero, atherosclerosis that represents high CVD risk can be present [11]. Phenotypic characterization of such patients is required in determining which of these can benefit from carotid ultrasound in an attempt to enhance their CVD stratification or/and more intensive cardiovascular risk factor management. In the present study, we therefore aimed to identify patient characteristics that can predict potential plaque presence in a relatively large cohort of women with RA and a concurrent low CVD risk according to the SCORE.

## Methods

### Patients

**Figure 1:**
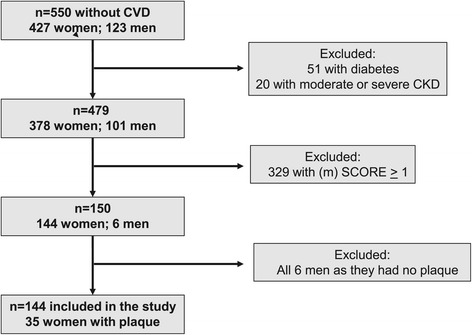
**Flowchart of patients included in the study.** CKD, chronic kidney disease; CVD, cardiovascular disease; (m) SCORE, (modified) Systematic COronary Risk Evaluation; n, number.

A total of 150 patients with RA and a SCORE of zero were recruited at the Hospital Universitario Marques de Valdecilla in Santander, Spain. As shown in Figure 1, participants originated in a group of 550 consecutive patients without established CVD (ischemic heart disease, cerebrovascular accident, peripheral arterial disease or/and heart failure) that underwent cardiovascular risk factor recording and carotid ultrasound as previously reported [11]. In the present study, we also excluded patients with type 1 diabetes, type 2 diabetes and moderate (estimated glomerular filtration rate = 30 to 59 ml/min/1.73 m^2^) or severe chronic kidney disease (estimated glomerular filtration rate <30 ml/min/1.73 m^2^) as these comorbidities represent high or very high CVD risk [8,11]. All patients in our ongoing study on CVD risk in RA meet the 1987 American College of Rheumatology [13] as well as the 2010 classification criteria for RA [14]. As all patients were enrolled at the same single center (see above), ethics’ approval was required and hence obtained from the Ethics Committee of Cantabria for Hospital Universitario de Valdecilla in Santander (Spain), and not from the other centers that some of the authors belonged to. Each participant gave written informed consent.

### Cardiovascular risk factors

Recorded patient characteristics are shown in Table 1. Conventional CVD risk factors were defined using previously reported methods by us [15]. Based on age, sex, smoking status, systolic blood pressure, total cholesterol and high-density lipoprotein (HDL) cholesterol concentrations, the SCORE was calculated to determine the 10-year risk of fatal CVD in a European population at low CVD risk, as recommended in persons living in Spain [8,11]. Since only patients with a SCORE of zero were included in the present study, calculation of the mSCORE as recommended by EULAR in RA patients [1], was not performed.

**Table 1:** **Recorded characteristics and their associations with plaque in 144 patients with a SCORE of zero**

	**Plaque**			
**Characteristics**	**Absent**	**Present**		
	**(n = 109)**	**(n = 35)**	**OR (95% CI)**	***P***
Demographic				
Age (years)	42.4 (9.5)	52.1 (5.7)	1.17 (1.09-1.25)	<**0.0001**
Conventional CV risk factors				
Hypertension	14.7	31.4	2.66 (1.09-6.53)	**0.01**
Systolic blood pressure (mmHg)	120 (13)	127 (16)	1.04 (1.01-1.07)	**0.01**
Diastolic blood pressure (mmHg)	76 (8)	78 (7)	1.04 (0.99-1.10)	0.09
Dyslipidemia	22.0	40.0	2.36 (1.04-5.37)	**0.04**
Total cholesterol (mmol/l)	5.0 (0.9)	5.5 (0.9)	1.80 (1.19-2.74)	**0.006**
LDL cholesterol (mmol/l)	2.9 (0.8)	3.2 (0.7)	1.01 (1.00-1.03)	**0.03**
HDL cholesterol (mmol/l)	1.7 (0.9-4.1)	1.7 (1.0-4.1)	1.01 (0.99-1.03)	0.4
Cholesterol-HDL cholesterol ratio	2.9 (1.5-5.7)	3.0 (1.9-5.7)	1.36 (0.85-2.18)	0.2
Triglycerides (mmol/l)	0.8 (0.4-2.2)	0.8 (0.4-4.3)	1.01 (1.00-1.02)	**0.04**
Triglyceride-HDL cholesterol ratio	1.0 (0.4-5.5)	1.1 (0.3-10.0)	1.28 (0.91-1.82)	0.1
Smoking ever	53.2	54.3	1.04 (0.48-2.26)	0.9
Current smoking	31.2	28.6	0.88 (0.38-2.05)	0.8
Body mass index (kg/m^2^)	24.4 (15.2-48.7)	24.7 (18.4-48.2)	1.06 (1.00-1.13)	0.06
Family history of CVD	25.2	8.6	1.03 (0.94-1.13)	0.6
Risk factor control				
Blood pressure <140/90 (mmHg)	89.9	74.3	0.32 (0.12-0.87)	**0.02**
LDL cholesterol <1.8 (mmol/l)	5.6	0	1.03 (0.88-1.21)	0.7
CVD drugs				
Antihypertensives	10.0	28.6	3.56 (1.35-9.41)	**0.01**
Statins	6.4	20.0	3.64 (1.17-11.36)	**0.02**
RA characteristics				
Age at disease onset (years)	34.4 (11.0)	43.3 (11.2)	1.08 (1.04-1.13)	**0.0002**
Duration (years)	5.5 (0.3-31.8)	4.8 (0.3-39.0)	1.01 (0.96-1.06)	0.6
RF and/or anti-CCP positive	60.6	45.7	0.55 (0.25-1.19)	0.1
Disease Activity Score in 28 joints	3.9 (4.7)	4.3 (7.3)	1.10 (0.82-1.48)	0.5
Joint erosion(s)	35.2	31.4	1.03 (0.87-1.24)	0.7
Extra-articular disease	15.6	14.3	0.79 (0.27-2.32)	0.7
C-reactive protein (mg/l)	1.0 (0.2-37.0)	1.0 (0.2-65.0)	1.02 (0.97-1.07)	0.4
Erythrocyte sedimentation rate (mm/hr)	10 (1-39)	8 (2-54)	1.00 (0.97-1.04)	0.8
Disease-modifying agents				
Synthetic				
Current use	97.2	100	2.55 (0.22-29.65)	0.5
Number ever used	2 (0-5)	2 (1-7)	1.46 (1.08-1.98)	**0.01**
Biologic				
Current use	42.2	34.3	0.72 (0.32-1.59)	0.4
Number ever used	0.6 (0.9)	0.8 (1.5)	1.15 (0.82-1.63)	0.4
Prednisone use ever	76.1	94.3	5.17 (1.15-23.32)	**0.03**
NSAID use ever	93.6	88.6	0.53 (0.44-1.96)	0.3
	**Plaque**			
**Characteristics**	**Absent**	**Present**		
	**(n = 94)**	**(n = 19)**	**OR (95% CI)**	***P***
EULAR modifier	1.1 (0.8)	0.9 (0.9)	0.80 (0.50-1.29)	0.4
Components^*^				
N = 0	24.8	40.0		
N = 1	49.5	31.5		
N = 2	20.2	20.0		
N = 3	5.5	8.5		

^*^Components include disease duration >10 years, rheumatoid factor or/and anti-cyclic citrullinated peptide positivity or/and extra-articular manifestations. Results are expressed as mean (SD), median (range) or proportion as appropriate. Data were analyzed in logistic regression models. Bold *P* values are significant SCORE, Systematic COronary Evaluation Score; n, number; OR, odds ratio; CI, confidence interval; CV, cardiovascular; LDL, low-density lipoprotein; HDL, high-density lipoprotein; CVD, cardiovascular disease; RA, rheumatoid arthritis; RF, rheumatoid factor; CCP, cyclic citrullinated peptide; NSAID, nonsteroidal anti-inflammatory drug; EULAR, European League Against Rheumatism.

Recorded cardiovascular drugs included antihypertensive agents and statins. Extra-articular manifestations comprised nodular disease, Felty’s syndrome, pulmonary fibrosis, rheumatoid vasculitis and secondary Sjogren’s syndrome [11].

### Carotid ultrasound

Arterial atherosclerotic plaques in the extracranial carotid tree were identified using the commercially available scanner, Mylab 70 Esaote (Genoa, Italy) equipped with a 7 to 12 MHz linear transducer and the automated software-guided technique radio frequency-Quality Intima Media Thickness in real-time (QIMT, Esaote, Maastricht, The Netherlands) was used [11]. Carotid artery plaque was identified as recommended in the Mannheim consensus, that is when a focal structure that encroaches into the arterial lumen of at least 0.5 mm or 50% of the surrounding intima-media thickness (IMT) value or demonstrates a thickness of >1.5 mm as measured from the media-adventitia interface of the intima-lumen interface, is present [16].

All assessments were made on the same day in each patient.

### Data analysis

Results are expressed as mean (standard deviation (SD)), median (range) or proportions as appropriate. The associations of recorded patient characteristics with plaque were first assessed using univariate logistic regression models; characteristics that were found to be related to atherosclerosis were subsequently entered in a multivariable stepwise backward conditional logistic regression model.

For characteristics that were independently associated with atherosclerosis, sensitivity versus false positive frequency (1-specificity) for predicting plaque presence was analyzed employing receiver operating characteristic (ROC) curves. The predictive accuracy of recorded characteristics was evaluated by the area under the curve (AUC). To determine the optimal cutoff value of baseline characteristics in predicting plaque presence, we calculated the Youden index using the following formula: sensitivity + specificity – 1, with the maximum obtained value corresponding to the optimal cutoff point [17]. We also calculated the negative and positive predictive values based on Bayes’ theorem as follows: positive predictive value = prevalence (p) x sensitivity/[p x sensitivity + (1 - p) × specificity] and negative predictive value = (1 - p) × specificity / [p x (1 - sensitivity) + (1 - p) × specificity] [18].

Statistical computations were made using the GB Stat program (Dynamic Microsystems, Inc., Silver Spring, MD, USA) and SPSS software, version 21 (IBM Corp., Armonk, NY, USA).

## Results

Of the initially recruited 150 patients with a SCORE of zero, 144 (96%) were women (Figure 1), 35 (24.3%) of whom had carotid artery plaque that was unilateral and bilateral in 21 (60%) and 14 (40%) of them, respectively. A carotid IMT of >0.900 mm was observed in only one (0.7%) patient who further had concurrent plaque. As none of the six participating men had carotid plaque, these were excluded from subsequent analysis. Amongst female participants, mean (SD) age and RA duration were 44.8 (11.7) and 8.2 (7.7) years, respectively. All except four patients had received synthetic disease-modifying antirheumatic drugs (DMARDs).

### Recorded characteristics in patients with and without carotid plaque

Table 1 gives potential CVD risk factors in patients with and without carotid plaque. Patients with carotid plaque were older and experienced later disease onset, sustained more prevalent hypertension and dyslipidemia and larger total and LDL cholesterol and triglyceride concentrations, and were more frequently treated with cardiovascular drugs. Blood pressure control was poorer in patients with carotid plaque, and none of them had adequate lipid control (LDL cholesterol <1.8 mmol/l) [8]. With regard to RA characteristics, patients with carotid plaque had larger prednisone and synthetic DMARD exposure. The number of EULAR modifier components was similar in patients with and without carotid plaque; two or three components were present in 28.5% and 25.2% of patients with and without plaque, respectively.

### Independent associations between recorded patient characteristics and carotid plaque

Table 2 shows the recorded characteristics that were independently related to plaque in a multivariable stepwise backward conditional logistic regression model. Of the seven characteristics entered into the model, two (systolic blood pressure and antihypertensive agent use) were deleted by backward elimination. Age, the number of synthetic DMARDs used and total cholesterol concentrations were independently associated with plaque. The sensitivity, specificity, positive and negative predictive value and correct prediction of the model were 51.4%, 91.7%, 66.7%, 85.5% and 81.9%, respectively. Replacement of the variable systolic blood pressure by hypertension in the model given in Table 2 did not alter the findings (data not shown).

**Table 2:** **Multivariable stepwise backward conditional logistic regression model for plaque**

**Characteristic**	**OR (95% CI)**	***P***
Age	1.15 (1.07-1.24)	<0.0001
Number of synthetic DMARDs used	1.51 (1.05-2.17)	0.03
Total cholesterol	1.66 (1.00-2.74)	0.04
Statin use	3.55 (0.93-13.52)	0.06
Prednisone use ever	3.77 (0.72-19.69)	0.1
Classification:		
Sensitivity, %	51.4	
Specificity, %	91.7	
Positive predictive value, %	66.7	
Negative predictive value, %	85.5	
Correct prediction, %	81.9	

The variables systolic blood pressure and antihypertensive agent use were deleted by backward elimination.

OR, odds ratio; CI, confidence interval; DMARDs, disease-modifying antirheumatic drugs.

**Figure 2:**
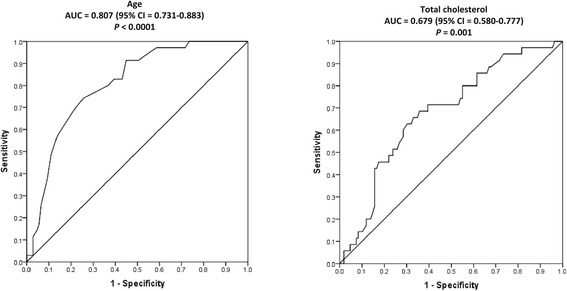
**Receiver operating characteristic (ROC) curves for predicting plaque presence by age and total cholesterol concentrations.** *P* values are given for the area under the curve (AUC)-plaque relations.

As shown in Figure 2, to estimate the accuracy of age and total cholesterol concentrations in predicting plaque presence, we performed ROC curve analysis. The AUC of the ROC curve was strongly associated with plaque for both characteristics (0.807 (*P* <0.0001) and 0.679 (*P* = 0.001), respectively).

To determine the optimal cutoff values for age and total cholesterol concentrations in determining plaque presence amongst patients not treated with cardiovascular drugs, we calculated the Youden index [17]. The obtained values and their corresponding sensitivity, specificity, positive and negative predictive values (as calculated from Bayes’ theorem [18]) are given in Table 3. The sensitivity, specificity and negative predictive value were 74%, 75% and 90% for age >49.5 years, and 63%, 70% and 86% for total cholesterol concentrations >5.4 mmol/l, respectively.

**Table 3:** **Optimal cutoff values for age and total cholesterol concentrations in ROC curves with corresponding characteristics amongst RA patients**

**Characteristics**	**Cutoff**	**Sensitivity**	**Specificity**	**PPV**	**NPV**
	**Value**	**(%)**	**(%)**	**(%)**	**(%)**
Age (years)	49.5	74	75	24	90
Total cholesterol (mmol/l)	5.4	63	70	22	86

ROC, receiver operating characteristic; RA, rheumatoid arthritis; PPV, positive predictive value; NPV, negative predictive value.

The low positive predictive values obtained in the current analysis (24% and 22% for age and total cholesterol, respectively) are expected as the overall plaque prevalence was, despite being clinically relevant, nevertheless numerically small at 24.3%. Further, this prevalence increased to 37.5% in patients (n = 80; 55.6%) with age or/and total cholesterol concentration of >49.5 years and 5.4 mmol/l, respectively; by contrast, the carotid plaque prevalence was only 7.8% in those (n = 64; 44.4%) with age or/and total cholesterol concentration of ≤49.5 years and 5.4 mmol/l, respectively.

Figure S1 in Additional file 1 shows the ROC analysis for the number of synthetic DMARDs used-carotid plaque relation. In contrast to age and total cholesterol concentrations, the number of synthetic DMARDs used was not significantly related to plaque in ROC analysis (AUC = 0.599 and corresponding *P* value = 0.08). The optimal cutoff value and its corresponding sensitivity, specificity and positive and negative predictive value were therefore not evaluated.

## Discussion

In this study, approximately one in four women with RA and a low predicted cardiovascular event rate as determined by SCORE, sustained atherosclerotic disease that represents very high CVD risk. In the population at large, men experience CVD events at a younger age than women, which is accounted for in the SCORE equation [8]. Congruently, 96% of our RA patients with a low calculated risk as estimated by SCORE were women. None of the six participating men had carotid plaque. Our findings show that low CVD risk as estimated by SCORE does not necessarily translate into absent high-risk atherosclerosis in women with RA. This supports recently reported evidence toward the need for improved CVD risk stratification in RA [3,4,6,7,11,12,15,19].

Our results indicate that among women with RA that are at low estimated CVD risk according to the SCORE, consideration of age and total cholesterol concentrations is useful in determining which of these patients experience high-risk subclinical atherosclerosis. Indeed, age >49.5 years and total cholesterol concentrations of >5.4% predicted plaque presence with a sensitivity and negative predictive value of 74% and 63% and 90% and 86%, respectively. Among patients aged >49.5 years or/and a total cholesterol concentration of >5.4 mmol/l, more than one-third had carotid plaque. Carotid artery plaque is independently associated with incident cardiovascular event rates amongst patients with RA. Whether incorporation of carotid ultrasound findings in CVD risk stratification strategies among RA patients with a low predicted CVD risk by SCORE, and particularly those aged >49.5 years or/and a total cholesterol concentration of >5.4 mmol/l, can reduce future cardiovascular event rates, should be assessed in future studies.

Persons that are at very high CVD risk as evidenced by carotid ultrasound-determined plaque presence, require intensive cardiovascular risk management [8]. Indeed, the LDL cholesterol target is <1.8 mmol/l in such cases [8]. In the present investigation, none of the patients with carotid plaque experienced adequate lipid control. In addition, 25.7% of our patients with carotid plaque had inadequate blood pressure control, a value that was larger than that in those without subclinical high-risk atherosclerosis, that is, 10.1%. Taken together, whereas the need for tight disease activity control has been amply documented, our findings also stress the notion that more stringent CVD risk factor control is required in at least a clinically relevant proportion of patients with RA that are at low estimated CVD risk according to SCORE.

RA characteristics or non-traditional rather than traditional CVD risk factors are reportedly most strongly associated with atherosclerosis in young patients with RA [12]. This contrasts to our findings, particularly among relatively young women with RA, as age and total cholesterol concentrations rather than RA characteristics were most consistently associated with plaque. This discrepancy could be due to rigorous RA activity control in the present cohort as the mean 28-item Disease Activity Score (DAS28) was within the range for low disease activity at 3.1 [20].

In this study, we report for the first time on potential predictors of plaque presence in patients with RA that are at low estimated CVD risk according to SCORE. Of interest in the present context, even amongst non-RA subjects, recent investigations have revealed a prevalence of carotid plaque as large as 34 to 59% despite an overall low estimated CVD risk as determined by the Framingham score [21,22]. Additionally, approximately 40% of persons with a low SCORE were found to experience multidetector computed tomography-determined coronary artery plaque that was further also related to traditional CVD risk factors [23].

Arts and colleagues [24] recently found that women with RA have disease activity-related reduced HDL-2 subfraction levels, which are reportedly more closely associated with CVD risk than total HDL cholesterol concentrations [25]. The role of decreased HDL-2 production in atherosclerosis among women with RA that have a low calculated risk as estimated by SCORE should be the subject of future studies.

Our data were derived from a single center and in white patients only. The findings in the present investigation require and merit validation in other population groups. In this regard, the prevalence of seropositivity for rheumatoid factor and anti-cyclic citrullinated peptide antibody was relatively low at 56.9%, which may have contributed to the lack of association of RA characteristics with carotid plaque. Notably also, at the time of our previous investigation on 370 consecutive patients [11], the plaque prevalence was only 13% amongst those with a SCORE value of zero. Upon investigating an additional 180 patients (see Figure 1 in the present manuscript), the respective prevalence has increased nearly twofold. We are uncertain why this occurred as our recruitment strategies have not been altered over time. Whereas the main purpose of the present study was to identify potential predictors of high-risk atherosclerosis in patients with low estimated CVD risk by SCORE, the increase in plaque prevalence over time among the respective cases in our setting nevertheless further reinforces the need for external validation.

## Conclusions

Approximately one-third of women with RA who experience a low SCORE value and are aged >49.5 years or/and have a total cholesterol concentration of >5.4 mmol/l experience high-risk atherosclerosis that requires intensive CVD risk management.

## Additional file

Additional file 1: Figure S1. Receiver operating characteristic (ROC) curves for predicting plaque presence by the number of disease-modifying agents used. *P* values are given for the area under the curve (AUC)-plaque relations.

## Abbreviations

AUC: area under the curve
CI: confidence interval
CVD: cardiovascular disease
DAS28: 28-item Disease Activity Score
DMARD: disease-modifying antirheumatic drugs
EULAR: European League Against Rheumatism
HDL: high-density lipoprotein
LDL: low-density lipoprotein
OR: odds ratio
RA: rheumatoid arthritis
ROC: receiver operating characteristic
(m) SCORE: (modified) Systematic COronary Risk Evaluation
SD: standard deviation
