# Supplementary figures and images for: Carotid artery plaque in women with rheumatoid arthritis and low estimated cardiovascular disease risk: a cross-sectional study
Source: Arthritis Res Ther. 2015 Mar 11;17(1):55. doi: 10.1186/s13075-015-0576-7 (PMC4376096; doi:10.1186/s13075-015-0576-7)

## Slide 1
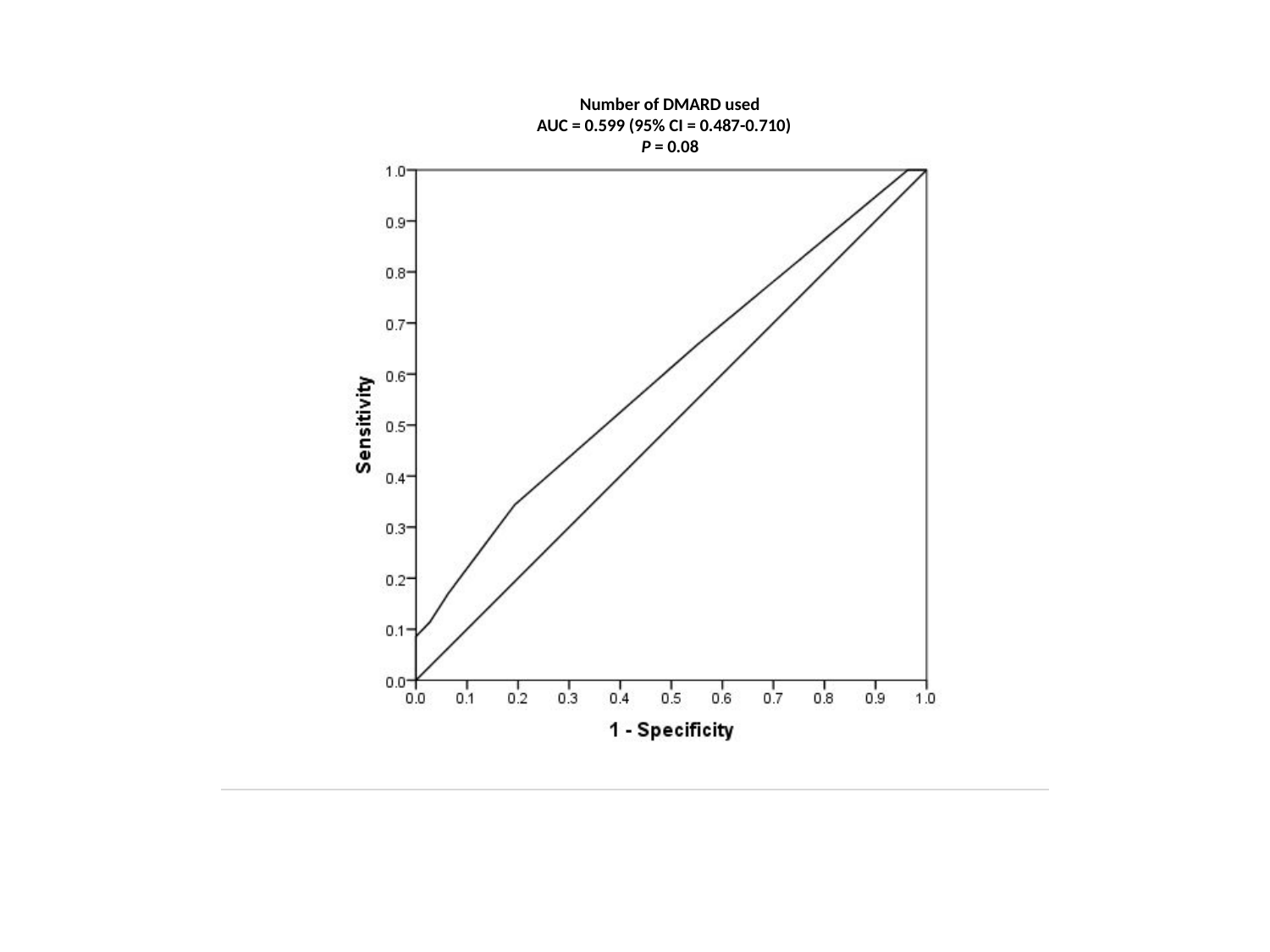

Number of DMARD used
AUC = 0.599 (95% CI = 0.487-0.710)
P = 0.08

Supplement: Additional file 1: Figure S1. — Receiver operating characteristic (ROC) curves for predicting plaque presence by the number of disease-modifying agents used. P values are given for the area under the curve (AUC)-plaque relations. [file 13075_2015_576_MOESM1_ESM.ppt]
